# Blindness in a Woman With Human Immunodeficiency Virus Infection and Syphilis

**DOI:** 10.1155/S1064744995000615

**Published:** 1995

**Authors:** Michael Luchi, Curtis Beauregard, Kevin Ault, Daniel Hinthorn

**Affiliations:** 1 Department of Internal Medicine University of Kansas Medical Center 6080-F 3901 Rainbow Boulevard Kansas KS 66160-7354 USA; 2 Department of Gynecology and Obstetrics University of Kansas Medical Center Kansas KS USA

## Abstract

*Background:* A concomitant infection with human immunodeficiency virus (HIV) may alter the natural history of other infections. Several reports indicate that syphilis may behave more aggressively when HIV infection is present.

*Case:* A woman presented with a rash involving her hands and feet and progressive loss of the vision in her right eye. Her serologic tests for syphilis and HIV infection were positive. A diagnosis of neurosyphilis was confirmed by an analysis of cerebrospinal fluid (CSF). She was treated with high-dose intravenous (IV) penicillin. Her skin lesions resolved, but her vision did not improve.

*Conclusion:* The incidence of HIV infection among women is rising. A patient with HIV and syphilis may develop neurosyphilis in a much shorter time than a patient without HIV infection.


Infectious Diseases in Obstetrics and Gynecology 3:198–201 (1995)
© 1996 Wiley-Liss, Inc.

### Blindness in a Woman With Human Immunodeficiency Virus Infection and Syphilis

**Michael Luchi, Curtis Beauregard, Kevin Ault, and Daniel Hinthorn**

*Departments of Internal Medicine (M.L., D.H.) and Gynecology and Obstetrics (C.B., K.A.), University of Kansas Medical Center, Kansas City, KS*

### Abstract

***Background:*** **A concomitant infection with human immunodeficiency virus (HIV) may alter the natural history of other infections. Several reports indicate that syphilis may behave more aggressively when HIV infection is present.**

***Case:*** **A woman presented with a rash involving her hands and feet and progressive loss of the vision in her right eye. Her serologic tests for syphilis and HIV infection were positive. A diagnosis of neurosyphilis was confirmed by an analysis of cerebrospinal fluid (CSF). She was treated with high-dose intravenous (IV) penicillin. Her skin lesions resolved, but her vision did not improve.**

***Conclusion:*** **The incidence of HIV infection among women is rising. A patient with HIV and syphilis may develop neurosyphilis in a much shorter time than a patient without HIV infection.** © 1996 Wiley-Liss, Inc.

Key words

Neurosyphilis, AIDS, sexually transmitted diseases

A 32-year-old woman presented to the ophthalmology clinic with progressive loss of the vision in her right eye and persistent, erythematous, lichenified plaques on the palms of her hands and the soles of her feet. Her visual loss had progressed insidiously over the past year without acute change. The lesions on her palms and soles had been present for 6 years, with intermittent exacerbations and partial remissions. A serum venereal disease research laboratory (VDRL) test was positive at a dilution of 1:256. She was admitted to the infectious diseases service.

Ten years before, the patient had been evaluated for a genital ulcer. A VDRL was negative. Three years later, she was treated for gonorrhea at a local health department clinic. Both VDRL and human immunodeficiency virus (HIV) serologies were negative. Four years before her admission, she had reported to the same clinic complaining of "spots" on her hands and feet that occasionally "peeled," loss of her eyebrows, and thinning hair. A VDRL was positive at a dilution of 1:128, and a test for antibodies to HIV was positive. She was treated for secondary syphilis with 2.4 million units of penicillin G benzathine. Despite counseling, she did not again seek medical attention until her presentation to the ophthalmology clinic.

At the time of her admission to the infectious diseases service, the patient appeared comfortable. Her temperature was 37.1°C. Her blood pressure was 100/70 and pulse was 114. There was only light perception in the right eye. The visual acuity in the left eye was 20/25. Bilateral vitreal haziness indicated active inflammation, but no vitreal cells. The retinas showed bilateral salt-and-pepper pigmentary retinitis and bone-spiculed peripheral pig-

Address correspondence/reprint requests to Dr. Michael Luchi, 6080-F, University of Kansas Medical Center, 3901 Rainbow Boulevard, Kansas City, KS 66160-7354.

Received July 21, 1995

Accepted November 15, 1995

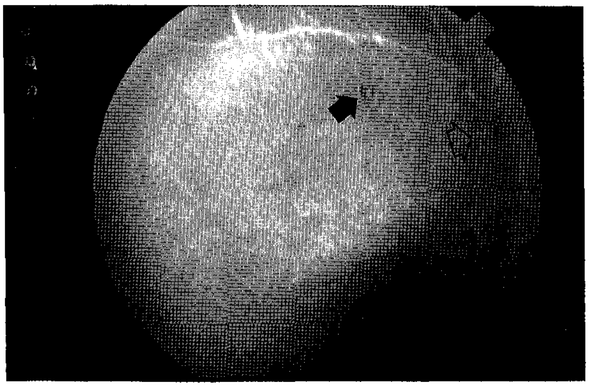

Fig. 1. Salt-and-pepper pigmentation of the retina with bony spiculation (solid arrow) and vascular sheathing (open arrow).

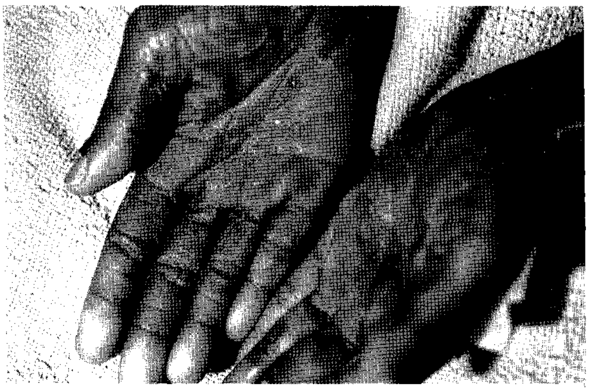

Fig. 2. Erythematous, keratotic plaques of the palms.

was no uveitis. A fungating lesion was present on the left buccal mucosa. There were bilateral, erythematous, keratotic plaques of the palms and soles (Fig. 2). Lymphadenopathy was present in the submandibular, axillary, and epitrochlear regions. The remainder of the general and neurologic examinations was unremarkable. A gynecologic evaluation was remarkable for a greenish vaginal discharge which was positive for *Trichomonas* sp. by wet-mount examination.

An analysis of cerebrospinal fluid (CSF) obtained by lumbar puncture revealed slightly turbid fluid with a WBC count of 9,640/μl (15% polymorphonuclear neutrophil leukocytes, 84% small mononuclear cells, and 1% large mononuclear cells). The glucose was low at 30 mg/dl, and the total protein was elevated at 156 mg/dl. The CSF VDRL was reactive at a dilution of 1:16. CSF cultures for bacteria, fungi, mycobacteria, and viral organisms were negative. Computed tomography of the head was normal. CBC, blood urea nitrogen, and creatinine were normal. Biopsies of the left palm and left buccal mucosa showed lichenoid psoriasiform dermatitis consistent with secondary syphilis. EIA and Western blot for HIV were positive. The CD4 count was 363/μl. A serum pregnancy test was negative.

A diagnosis of neurosyphilis was made, and the patient was treated with 2 million units (mIU) of intravenous (IV) penicillin G q 4 h for 10 days. After 9 days of therapy, a repeat CSF analysis showed a marked decline in WBCs (190/μl, 100% small mononuclear cells) and total protein (78 mg/dl). The glucose remained low at 38 mg/dl. The VDRL was essentially unchanged at 1:8. Six weeks after she had completed therapy, the CSF WBC count was 39/μl (100% small mononuclear cells), with a total protein of 49 mg/dl and a normal glucose of 46 mg/dl. The CSF VDRL had declined to 1:2. The serum VDRL was 1:128. Trimethoprim/sulfamethoxazole was begun for prophylaxis of *Pneumocystis carinii* pneumonia at the time of discharge. While the patient was on zidovudine, 500 mg/day, her CD4 count rose to 520/μl. Her rash and oral lesions resolved. Her vision was unimproved and her ophthalmological examination was unchanged, which was interpreted as irreversible damage to the retina, not ongoing infection. Therefore, additional anti-infective therapy was not administered.

### DISCUSSION

This case of neurosyphilis in an HIV-seropositive woman underscores important trends in the changing epidemiology of sexually transmitted diseases, serves as an example of the aggressive nature of syphilis in HIV-infected individuals, and highlights important questions in the treatment of syphilis when there is concomitant HIV infection.

The incidence of syphilis rose sharply in the mid-1980s, reversing a decline which had begun in the 1940s. In women, the incidence of syphilis has abated considerably since then, but has remained significantly above the nadir achieved in the late 1970s and the 1980s. The incidence of HIV infection among women has risen steadily in recent years. In 1994, 18% of all acquired immunodeficiency syndrome (AIDS) cases occurred in females >12 years of age, nearly 3-fold greater than the proportion reported for 1985. AIDS is now the fourth leading cause of death among women aged

25–44 years in the United States.[2] Two-thirds of the women with AIDS report having had sex with at-risk partners, and over one-quarter have histories of injecting-drug use.[3] The proportion of heterosexually acquired cases of AIDS has tripled from 2% in 1981 to 7% in 1993. Thus, the epidemiologic trends of HIV infection in the United States are shifting dramatically. Although AIDS was once considered to be an illness afflicting almost exclusively young homosexual men, a substantial proportion of its victims are now women infected through heterosexual intercourse or injecting-drug use.

Given the increasing prevalence of both of these infections, one might expect that the rate of coinfection is also rising. Indeed, the presence of syphilis is considered a risk factor for HIV infection.[4] Precise data on the number of men and women who are coinfected with syphilis and HIV are not available, but several reports clearly indicate that coinfection is becoming more prevalent.[5–8]

It has also become apparent that the pathobiology of syphilis in HIV-infected patients differs from that in immunocompetent patients in several ways. Neurosyphilis appears to occur both more frequently and with a shorter latency in HIV-infected individuals. In untreated immunocompetent individuals, the progression to neurosyphilis occurs from 5 to 30 years after infection with syphilis. In one series of HIV-infected patients, however, more rapid progression was suggested both by the relatively young age (mean 37 years) of the patients and by its rapid onset.[7] In this series, 2 patients developed neurosyphilis within 6 months of receiving the standard treatment for latent syphilis. Similarly, in a series of patients from Atlanta, the median interval between the last dose of penicillin and the diagnosis of neurosyphilis was 8 months.[8] This rapid progression occurs even among HIV-infected patients who possess relatively good immune function by current standards. In most patients with HIV infection, opportunistic infections, such as pneumonia caused by *Pneumocystis carinii*, generally do not occur before the CD4 count drops to $<200/\mu l$.[9] The patients in the Atlanta series had a median CD4 count of $344/\mu l$ (similar to that of our patient), and 7/11 patients had CD4 counts $>200/\mu l$ (range 23–972/μl).[8]

In AIDS patients, early neurosyphilis affects mesodermal structures such as the meninges and blood vessels and may cause asymptomatic illness, meningitis, cranial-nerve abnormalities (most commonly the second and eighth nerves[6]), and stroke. Ophthalmologic disease, as described in our patient, is common, manifested as uveitis, retinitis, or retrobulbar neuritis.[6–8,10] Ocular symptoms include blurred vision and blindness.[6] These characteristics stand in contrast to those of late neurosyphilis as described in the preantibiotic era. In these cases, the direct involvement of neurons leads to the classic manifestations of tabes dorsalis, general paresis, and dementia.[5]

The traditional classification of syphilis distinguishes the secondary stage of illness, most commonly presenting as a rash, from the tertiary phase (neurosyphilis and cardiovascular and gummatous syphilis), in which rash is uncommon. This classification may not apply in the presence of HIV infection. As in our case, 4/11 patients from the Atlanta series had dermatologic manifestations of secondary syphilis at the time they were diagnosed with neurosyphilis. Their lesions were typically papulosquamous or maculopapular rashes involving the palms or soles. Patchy alopecia was also seen.[8] Thus, in HIV-infected patients, lesions of secondary syphilis may be a sign of much more extensive and serious disease.

A common theme in the literature of syphilis and HIV infection is the progression to neurosyphilis despite appropriate treatment at the time of the original diagnosis of syphilis.[7,8,10] The reason for the ineffectiveness of treatment is not clear, but likely results from the inability of the HIV-infected host to eradicate the infecting organisms.[5] Obviously, the previously recommended regimen of benzathine penicillin G [a single intramuscular (IM) dose of 2.4 mIU] for the treatment of secondary syphilis in HIV-infected patients does not reliably eradicate treponemes from the CSF,[11] probably because penicillin does not reach detectable concentrations in the CSF when administered in this form.[5] In the Atlanta series, in contrast, after the patients' treatment with high-dose penicillin G (3–4 mIU IV q 4 h for 10 days), treponemes were no longer detectable in the 3 patients who had positive pretreatment CSF.[8] Despite high-dose IV penicillin, 1 patient in this series developed a stroke secondary to meningovascular syphilis within 2 months of completing the therapy. Thus, even the most intense of previously recommended regimens does not ensure that the disease progression will be interrupted.

In the absense of large clinical trials, there is considerable uncertainty about what the proper evaluation and treatment for patients with syphilis and HIV infection should be. Certainly, the presence of one infection should always prompt testing for the other. For early (primary or secondary) syphilis with coexisting HIV infection, some experts recommend empiric therapy of either 3 doses of benzathine penicillin, 2.4 mIU IM given at weekly intervals, or 10 daily doses of 1.2 mIU of procaine penicillin.[5] The current guidelines from the Centers for Disease Control and Prevention (CDC) continue to recommend 1 dose of 2.4 mIU of benzathine penicillin for either condition.[12] For latent syphilis, the CDC recommends 3 doses of benzathine penicillin. We prefer the more aggressive approach to therapy.

Experts differ on the need for a CSF examination in a patient with HIV infection and early syphilis. Some recommend empiric treatment[5] and others recommend routine analysis of the CSF.[11] However, there is uniform agreement that a patient with latent syphilis or any evidence of neurologic abnormality should have an examination of the CSF. Patients in any stage of syphilis require extremely close follow-up to be certain that the serum VDRL declines appropriately. For a patient with documented neurosyphilis, repeat CSF examinations are required. Interestingly, the initial CSF WBC count in our patient was considerably higher than that reported in the Atlanta series (9,640/μl vs. a mean of 38/μl with a range of 4–210/μl). With such a high CSF WBC count, a turbid sample would be expected. It was described as only "slightly turbid," raising the strong possibility that the initial CSF WBC was incorrect.

In conclusion, our case exemplifies the rising incidence of syphilis and HIV infection among women, the aggressive nature of syphilis in HIV-infected patients, and the difficulty in treating patients with these 2 infections. Syphilologists agree that such a patient should be followed closely after treatment to be certain that the serum VDRL declines appropriately and that, at the very least, a strong index of suspicion should be maintained for neurosyphilis, even after the patient has been treated. The precise treatment for a patient with early syphilis and the need for an examination of the CSF remain areas for investigation.

### ACKNOWLEDGMENTS

We thank Dr. Hasan Hakim of the Department of Ophthalmology for assistance in obtaining the photograph of the patient's fundus.

### REFERENCES

1. Division of STD/HIV Prevention: Sexually Transmitted Disease Surveillance, 1993. U.S. Department of Health and Human Services, Public Health Service. Atlanta: Centers for Disease Control and Prevention, December 1994.
2. Update: AIDS among Women—United States, 1994. MMWR 44, 1995.
3. Heterosexually acquired AIDS—United States, 1993. MMWR 43, 1994.
4. Quinn TC, Cannon RO, Glasser D, et al.: The association of syphilis with risk of human immunodeficiency virus infection in patients attending sexually transmitted disease clinics. Arch Intern Med 150:1297, 1990.
5. Musher DM: Syphilis, neurosyphilis, penicillin, and AIDS. J Infect Dis 163:1201, 1991.
6. Musher DM, Hamill RJ, Baughn RE: Effect of human immunodeficiency virus (HIV) infection on the course of syphilis and on the response to treatment. Ann Intern Med 113:872, 1990.
7. Katz DA, Berger JR: Neurosyphilis in acquired immunodeficiency syndrome. Arch Neurol 46:895, 1989.
8. Gordon SM, Eaton ME, George R, et al.: The response of symptomatic neurosyphilis to high-dose intravenous penicillin G in patients with human immunodeficiency virus infection. N Engl J Med 331:1469, 1994.
9. Walzer PD: *Pneumocystis carinii*. In Mandell GL, Bennett JE, Dolin R (eds): Mandell, Douglas, and Bennett's Principles and Practice of Infectious Diseases. 4th ed. New York: Churchill Livingstone, p 2475, 1995.
10. Johns DR, Tierney M, Felsenstein D: Alteration in the natural history of neurosyphilis by concurrent infection with the human immunodeficiency virus. N Engl J Med 316:1569, 1987.
11. Lukehart SA, Hook EW, Baker-Zander SA, Collier AC, Critchlow CW, Handsfield HH: Invasion of the central nervous system by *Treponema pallidum:* Implications for diagnosis and treatment. Ann Intern Med 109:855, 1988.
12. Centers for Disease Control and Prevention: 1993 Sexually transmitted diseases treatment guidelines. MMWR 42(RR-14):27–37, 1993.
